# Supplementary material for: Targeted ortholog search in unannotated genome assemblies with fDOG-Assembly
Source: Nucleic Acids Res. 2026 Sep 28;54(18):gkag929. doi: 10.1093/nar/gkag929 (PMC13617353; doi:10.1093/nar/gkag929)
Supplement: Supplementary file 1 [file gkag929_supplemental_files.zip › Supplementary_Figure_S10_Muelbaier_fDA.pdf]

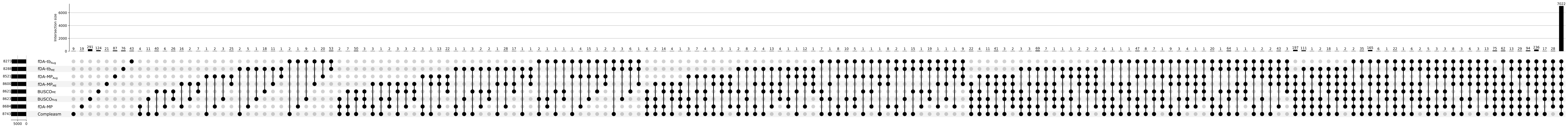
Intersection size
fDA-tb$_{Aug}$
fDA-tb$_{Ref}$
fDA-MP$_{Aug}$
fDA-MP$_{Ref}$
BUSCO$_{Ref}$
BUSCO$_{Aug}$
fDA-MP
Compleasm
