# Supplementary material for: Targeted ortholog search in unannotated genome assemblies with fDOG-Assembly
Source: Nucleic Acids Res. 2026 Sep 28;54(18):gkag929. doi: 10.1093/nar/gkag929 (PMC13617353; doi:10.1093/nar/gkag929)
Supplement: Supplementary file 1 [file gkag929_supplemental_files.zip › Supplementary_material_Muelbaier_fDA_r2.v0.pdf]

Supplementary material

SUPPLEMENTARY MATERIAL 1

Supplementary Tables 2
Table S1. Cross-reference table BUSCO ID to UniProt ID 2
Table S2. Precomputed Augustus models used for gene prediction with fDA-tb_{Aug}, fDA-MP_{Aug} and $BUSCO_{Aug}$ 2
Table S3. Primer species used in the core ortholog compilation for 5,000 human proteins 2
Table S4. Primer taxa for compiling the core orthologous groups for the beta-lactam biosynthetic genes 3
Table S5. Proteins involved in beta-lactam biosynthesis used as seeds for the ortholog search with fDOG and fDOG-Assembly 4
Table S6. Ortholog predictions downloaded from the QfO benchmarking server 4
Table S7. Number of inferred orthologs per search species and corresponding benchmarking run 4
Table S8. Gaps in the phylogenetic profiles of the Metazoa odb10 dataset resolved per species 5
Table S9. Number of orthology assignments of fDA for set 2 6
Table S10. Performance of fDA in recovering human – Nematostella co-orthologous relationships predicted by InParanoid 6
Table S11. Soil invertebrate species used for beta-lactam gene search with fDA 6
Table S12. Assignment of soil invertebrate species to clusters of β-lactam biosynthesis repertoires 6
Table S13. Contamination screen of soil invertebrate orthologs to β-lactam biosynthesis genes 6
Table S14. Extended contamination screen for cefE and cefF considering genomic neighbourhood 6

Supplementary Data 7
Supplementary Data S1. Core orthologous groups used in the fDA runs 7
Supplementary Data S2. Phylogenetic profiles for beta-lactam biosynthesis factors 7

Supplementary Figures 8
Supplementary Figure S1. Mapping of UniProt-IDs to fDA gene predictions. 8
Supplementary Figure S2. Agreement between the $BUSCO_{Aug}$ gene annotation and the pre-annotated genes in the individual QfO reference species. ‘ 9
Supplementary Figure S3. Agreement between the $BUSCO_{ME}$ gene annotation and the pre-annotated genes in the individual QfO reference species. 9
Supplementary Figure S4. Agreement between the fDA-tb_{Aug} gene annotation and the pre-annotated genes in the individual QfO reference species. 10
Supplementary Figure S5. Agreement between the fDA-tb_{ME} gene annotation and the pre-annotated genes in the individual QfO reference species. 10
Supplementary Figure S6. Agreement between the fDA-MP gene annotation and the pre-annotated genes in the individual QfO reference species. 11
Supplementary Figure S7. Agreement between the fDA-MP_{Aug} gene annotation and the pre-annotated genes in the individual QfO reference species. 11
Supplementary Figure S8. Agreement between the fDA-MP_{ME} gene annotation and the pre-annotated genes in the individual QfO reference species. 12
Supplementary Figure S9. Agreement between the Compleasm gene annotation and the pre-annotated genes in the individual QfO reference species. 12
Supplementary Figure S10. Overlap of the ortholog assignments made by fDA, Compleasm and BUSCO. 13
Supplementary Figure S11. Consistency of orthology assignments from assembly-based tools with those from proteome-based tools. 13
Supplementary Figure S12. The fDA-tb_{AUG} ortholog to the BUSCO gene 613851at33208 overlaps with a curated NCBI RefSeq gene that is not included in the QfO reference proteome. 13
Supplementary Figure S13. Phylogenetic Profiles of genes involved in beta-lactam biosynthesis across soil-living invertebrates. 14
Supplementary Figure S14. Domain Architecture of pcbAB (d-(L-a-Aminoadipyl)-L-Cysteinyl-D-Valine Synthetase) from Streptomyces clavuligerus. 14

Supplementary Tables

Table S1. Cross-reference table BUSCO ID to UniProt ID

See *separate file Supplementary_Table_1-Muelbaier-fDA.xlsx*

Table S2. Precomputed Augustus models used for gene prediction with fDA-tb$_{Aug}$, fDA-MP$_{Aug}$ and BUSCO$_{Aug}$

Genome assembly	Augustus model
Caenorhabditis elegans	caenorhabditis
Danio rerio	zebrafish
Drosophila melanogaster	fly
Gallus gallus	chicken
Helobdella robusta	caenorhabditis
Ixodes scapularis	fly
Nematostella vectensis	fly
Rattus norvegicus	human
Tribolium castaneum	tribolium2012
Xenopus tropicalis	human

Table S3. Primer species used in the core ortholog compilation for 5,000 human proteins

UniprotID	Species
UP000007062	*Anopheles gambiae* (African malaria mosquito)
UP000000589	*Mus musculus* (Mouse)
UP000005640	*Homo sapiens* (Human)
UP000009136	*Bos taurus* (Bovine)
UP000001038	*Oryzias latipes* (Japanese rice fish)
UP000002280	*Monodelphis domestica* (Gray short-tailed opossum)
UP000001519	*Gorilla gorilla gorilla* (Western lowland gorilla)
UP000002254	*Canis lupus familiaris* (Dog)
UP000002277	*Pan troglodytes* (Chimpanzee)
UP000001554	*Branchiostoma floridae* (Florida lancelet)
UP000018468	*Lepisosteus oculatus* (Spotted gar)

Table S4. Primer taxa for compiling the core orthologous groups for the beta-lactam biosynthetic genes

Species	NCBI ID	RefSeq ID	Identifier
Amycolatopsis albispora	1804986	GCF_003312875.1	AMYAL@1804986@003312875_1
Amycolatopsis magusensis	882444	GCF_017875555.1	AMYMA@882444@017875555_1
Aspergillus nidulans	227321	GCF_000149205.2	ASPNI@227321@000149205_2
Aspergillus puulaauensis	1220207	GCF_016861865.1	ASPPU@1220207@016861865_1
Hyaloscypha bicolor	1095630	GCF_002865645.1	HYABI@1095630@002865645_1
Modestobacter excelsi	2213161	GCF_005930495.1	MODEX@2213161@005930495_1
Streptantibioticus cattleyicolor	1003195	GCF_000237305.1	STRCA@1003195@000237305_1
Streptomyces clavuligerus	1901	GCF_000148465.1	STRCL@1901@000148465_1
Streptomyces jumonjinensis	1945	GCF_009600885.1	STRJU@1945@009600885_1
Streptomyces katsurahamanus	2577098	GCF_009600895.1	STRKA@2577098@009600895_1
Streptomyces megasporus	44060	GCF_000718985.1	STRME@44060@000718985_1
Streptomyces odonnellii	1417980	GCF_000981895.1	STROD@1417980@000981895_1
Streptomyces sulfonofaciens	68272	GCF_014656295.1	STRSU@68272@014656295_1
Streptomyces tirandamycinicus	2174846	GCF_003097515.1	STRTI@2174846@003097515_1
Trichophyton benhamiae	663331	GCF_000151125.1	TRIBE@663331@000151125_1
Trichophyton rubrum	559305	GCF_000151425.1	TRIRU@559305@000151425_1
Trichophyton verrucosum	663202	GCF_000151505.1	TRIVE@663202@000151505_1

Table S5. Proteins involved in beta-lactam biosynthesis used as seeds for the ortholog search with fDOG and fDOG-Assembly

Gene Name	Reference species	NCBI taxonomy ID	Uniprot ID	NCBI Accession
**cefE**	*Streptomyces clavuligerus*	1901	P18548	WP_003952493.1
**cefF**	*Streptomyces clavuligerus*	1901	P42220	WP_003952498.1
**cmcH**	*Streptomyces clavuligerus*	1901	O85728	WP_003952499.1
**cmcI**	*Streptomyces clavuligerus*	1901	B5GLB3	WP_003952496.1
**cmcJ**	*Streptomyces clavuligerus*	1901	B5GLB4	WP_003952497.1
**cefD**	*Streptomyces clavuligerus*	1901	P18549	WP_003952494.1
**pcbC**	*Streptomyces clavuligerus*	1901	P10621	WP_003952506.1
**pcbAB**	*Streptomyces clavuligerus*	1901	Q01757	WP_003952505.1
**penDE**	*Aspergillus nidulans*	162425	P21133	XP_660227.1

Table S6. Ortholog predictions downloaded from the QfO benchmarking server

Dataset
BBH
Domainoid+
EnsemblCompara
Hieranoid2
InParanoid5
MethaPhOrs v2.5
OMA pairs
OrthoFFGC
OrthoFinder 2.5.5
OrthoInspector 3.5
PANTHER 18.0 all
RSD
SonicParanoid sens

Table S7. Number of inferred orthologs per search species and corresponding benchmarking run

	fDA-tb		fDA-MP			BUSCO		Compleasm
	Aug	ME	Aug	ME	MP	Aug	ME	MP

C. elegans	728	735	690	636	646	741	739	729
D. rerio	1164	1169	1158	1163	1165	1245	1156	1155
D. melanogaster	926	940	958	958	966	960	961	957
G. gallus	897	918	909	906	908	914	917	918
H. robusta	803	870	915	947	939	895	972	921
I. scapularis	863	971	961	1049	982	894	919	928
N. vectensis	872	1049	944	1019	1007	935	957	948
R. norvegicus	1009	1073	1015	1029	1045	1020	1276	1060
T. castaneum	945	952	955	967	961	959	960	955
X. tropicalis	949	975	952	955	952	958	958	958
**Total**	**9156**	**9652**	**9457**	**9629**	**9571**	**9521**	**9815**	**9529**

Table S8. Gaps in the phylogenetic profiles of the Metazoa odb10 dataset resolved per species

	**Gaps$**
Gallus gallus	32
Rattus norvegicus	5
Tribolium castaneum	4
Caenorhabditis elegans	55
Nematostella vectensis	26
Ixodes scapularis	43
Danio rerio	1
Helobdella robusta	21
Xenopus tropicalis	14
Drosophila melanogaster	13
**Total**	**214**

$ We only consider gaps where 9 of the 10 species are represented by an ortholog

Table S9. Number of orthology assignments of fDA for set 2

	Rat	*N. vectensis*	Total
	#orthologs (#mapped to UniProt)	#orthologs (#mapped to UniProt)	#orthologs (#mapped to UniProt)
**fDA-tb$_{Aug}$**	5,749 (4,920)	3,246 (2,702)	8,995 (7,622)
**fDA-tb$_{ME}$**	5,642 (4,781)	4,365 (3,441)	10,007 (8,222)
**fDA-MP$_{Aug}$**	5,104 (4,653)	2,318 (1,941)	9,468 (6,594)
**fDA-MP$_{ME}$**	5,442 (4,634)	2,821 (2,190)	8,263 (6,824)
**fDA-MP**	5,021 (4,703)	2,434 (2,119)	7,455 (6,822)

Table S10. Performance of fDA in recovering human – Nematostella co-orthologous relationships predicted by InParanoid

**Group layout**		**1:1**	**1:n**	**n:1**	**n:n**
		**Orthology assignments: #groups (#pairs)**			
N. vectensis	InParanoid	1425 (1425)	167 (527)	145 (334)	59 (1280)
	fDA-tb$_{Aug}$	762 (762)	76 (115)	84 (156)	24 (114)
	fDA-tb$_{ME}$	1117 (1117)	105 (200)	107 (202)	29 (143)
	fDA-MP$_{Aug}$	797 (797)	73 (89)	95 (181)	21 (56)
	fDA-MP$_{ME}$	1032 (1032)	90 (127)	113 (221)	22 (62)
	fDA-MP	1023 (1023)	91 (224)	113 (123)	23 (75)

Table S11. Soil invertebrate species used for beta-lactam gene search with fDA

See Supplementary_Table_11_Muelbaier_fDA.xlsx

Table S12. Assignment of soil invertebrate species to clusters of β-lactam biosynthesis repertoires

See Supplementary_Table_12_Muelbaier_fDA.xlsx

Table S13. Contamination screen of soil invertebrate orthologs to β-lactam biosynthesis genes

See Supplementary_Table_13_Muelbaier_fDA.xlsx

Table S14. Extended contamination screen for cefE and cefF considering genomic neighbourhood

See Supplementary_Table_14_Muelbaier_fDA.xlsx

Supplementary Data

Supplementary Data S1. Core orthologous groups used in the fDA runs

See Supplementary_Data_1_core-orthologous-groups.tar.gz

Supplementary Data S2. Phylogenetic profiles for beta-lactam biosynthesis factors

See Supplementary_Data_2_fDA-output.tar.gz

Supplementary Figures

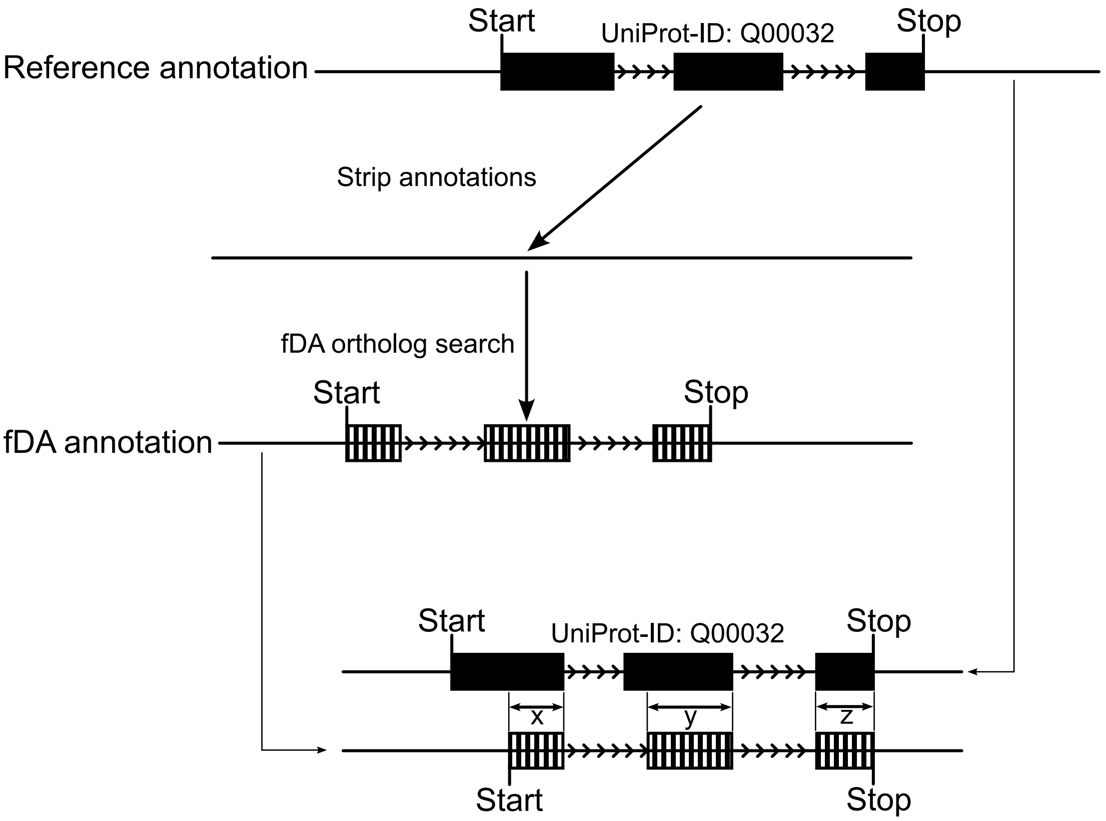

Supplementary Figure S1. Mapping of UniProt-IDs to fDA gene predictions. Reference gene annotations are stripped from the assembly. The assembly is then used for an fDA ortholog search. To assign the ortholog identified by fDA a UniProt-ID, we compare the genomic positions of the corresponding exons to those in the reference annotation and determine the overlap (x, y, and z in the figure). If the two gene annotations reside on the same strand, and if the sum of the overlaps exceeds 0.5 x length of the reference gene, we transfer the UniProt-ID of the corresponding protein to that encoded by the fDA annotated gene. See Supplementary Figs. S2 – S8 for a distribution of overlaps for the different ortholog search tools and search modes.

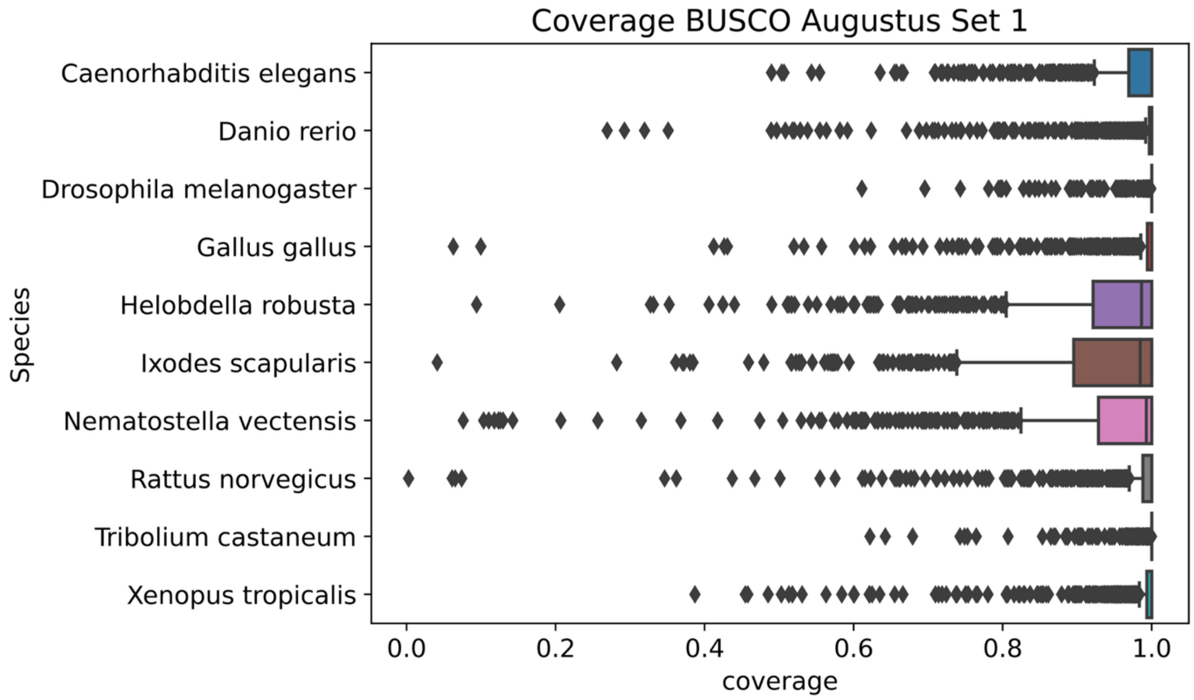

Supplementary Figure S2. Agreement between the BUSCO$_{Aug}$ gene annotation and the pre-annotated genes in the individual QfO reference species. 'Coverage' denotes the fraction of nucleotides in the protein-coding exons of the pre-annotated genes that are contained in protein-coding exons of the BUSCO-annotated genes (see Supplementary Figure S1 for details). The coverage distribution is provided for the genes in benchmark set 1.

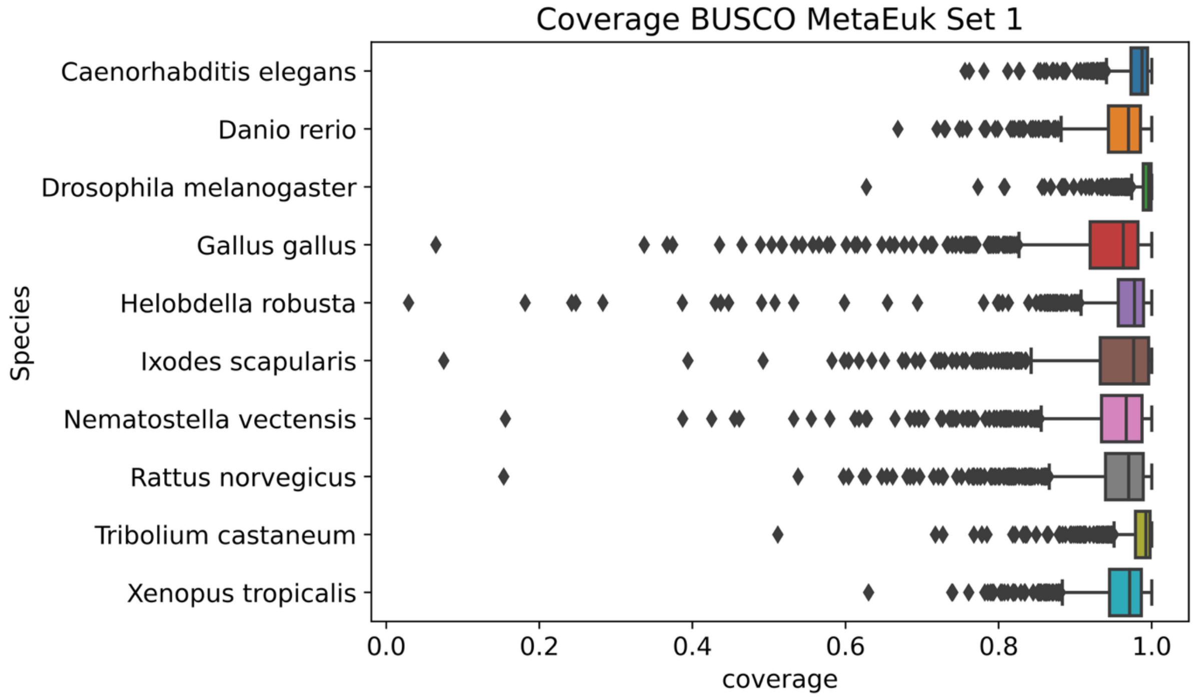

Supplementary Figure S3. Agreement between the BUSCO$_{ME}$ gene annotation and the pre-annotated genes in the individual QfO reference species. 'Coverage' denotes the fraction of nucleotides in the protein-coding exons of the pre-annotated genes that are contained in protein-coding exons of the BUSCO$_{ME}$-annotated genes (see Supplementary Figure S1 for details). The coverage distribution is provided for the genes in benchmark set 1.

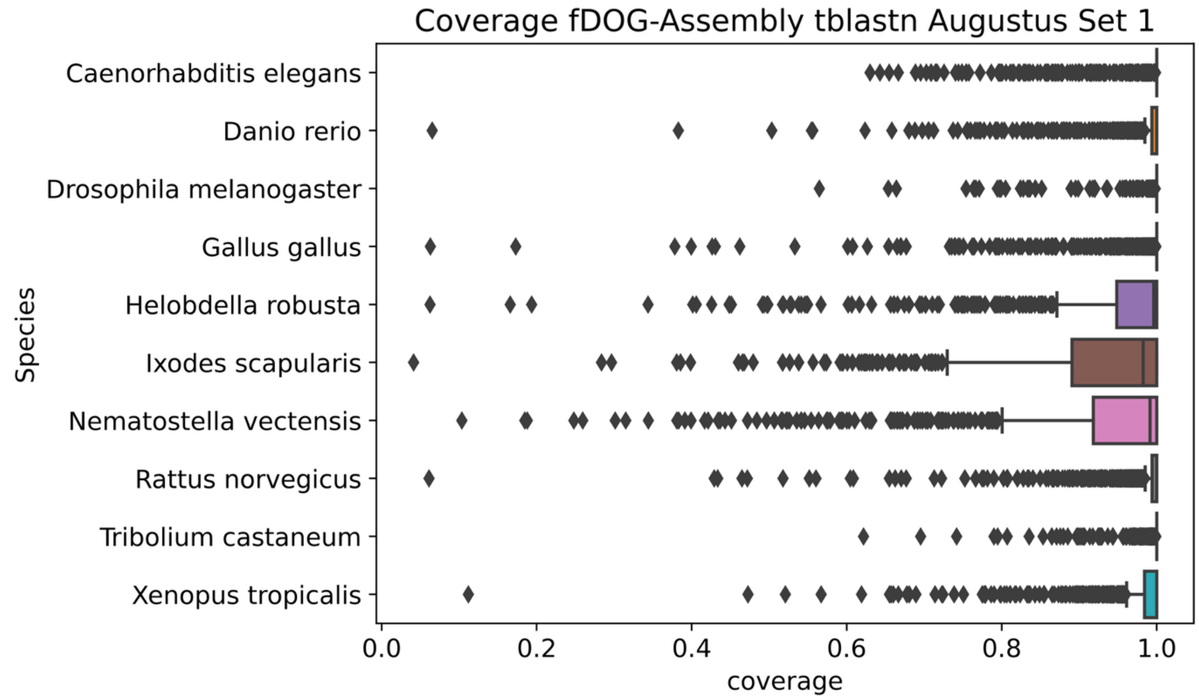

Supplementary Figure S4. Agreement between the fDA-tb$_{Aug}$ gene annotation and the pre-annotated genes in the individual QfO reference species. 'Coverage' denotes the fraction of nucleotides in the protein-coding exons of the pre-annotated genes that are contained in protein-coding exons of the fDA-tb$_{Aug}$-annotated genes (see Supplementary Figure S1 for details). The coverage distribution is provided for the genes in benchmark set 1.

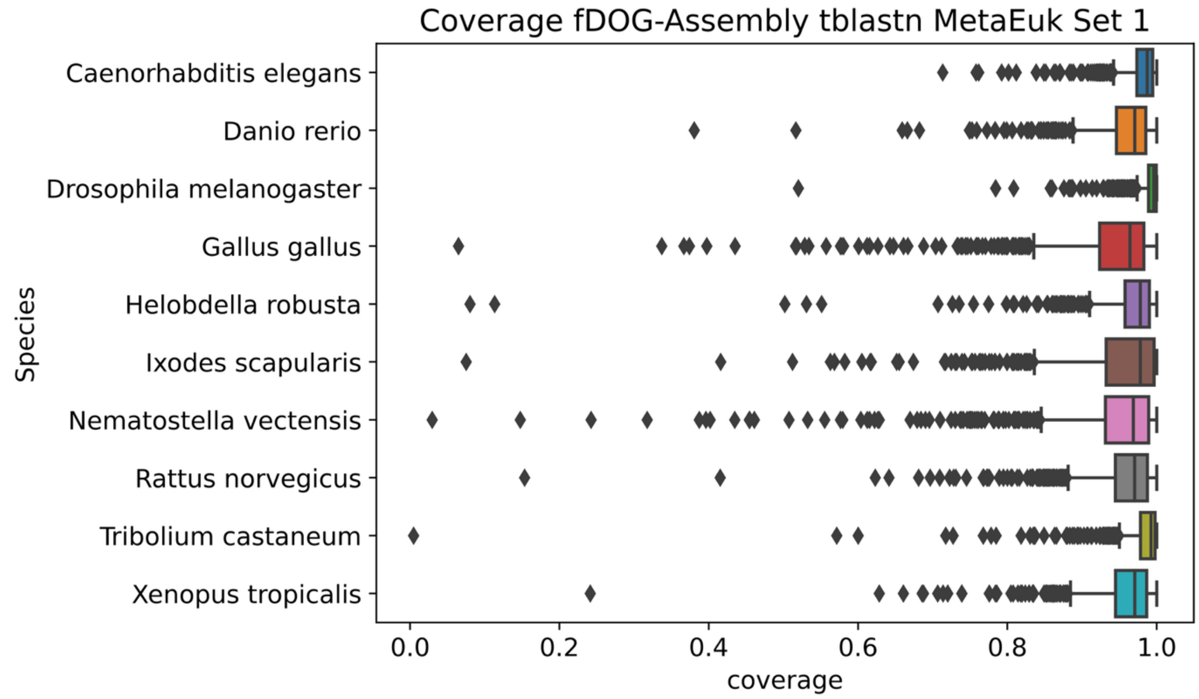

Supplementary Figure S5. Agreement between the fDA-tb$_{ME}$ gene annotation and the pre-annotated genes in the individual QfO reference species. 'Coverage' denotes the fraction of nucleotides in the protein-coding exons of the pre-annotated genes that are contained in protein-coding exons of the fDA-tb$_{ME}$-annotated genes (see Supplementary Figure S1 for details). The coverage distribution is provided for the genes in benchmark set 1.

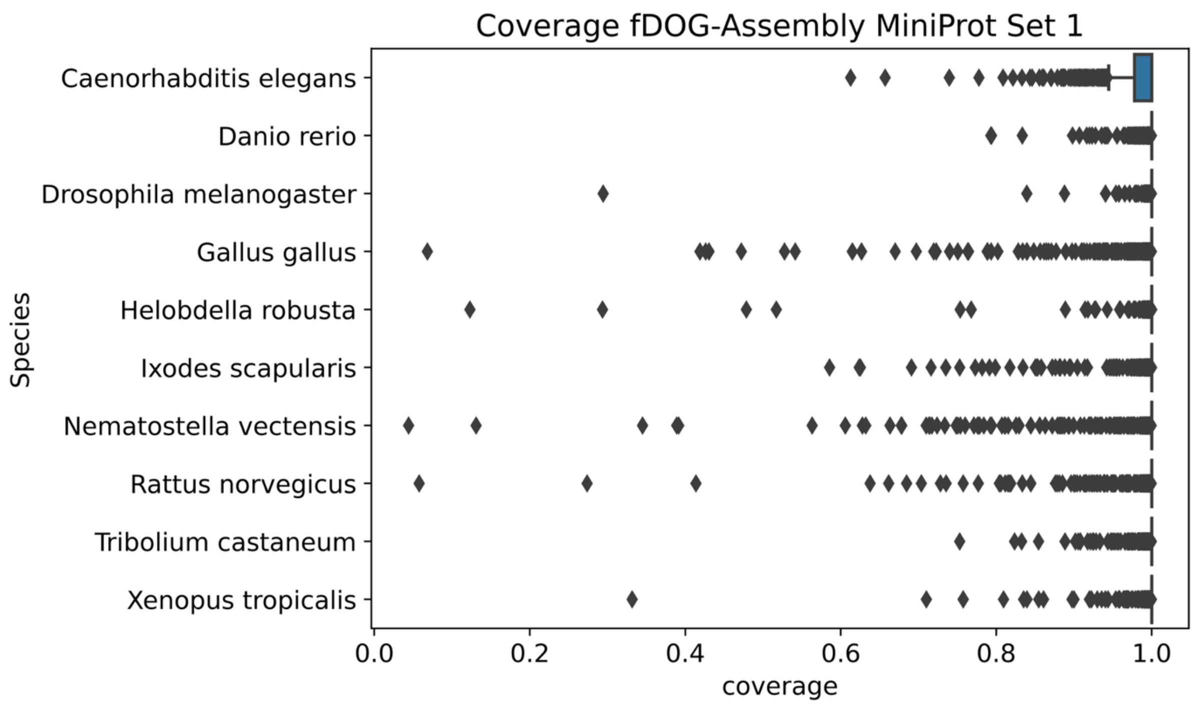

Supplementary Figure S6. Agreement between the fDA-MP gene annotation and the pre-annotated genes in the individual QfO reference species. 'Coverage' denotes the fraction of nucleotides in the protein-coding exons of the pre-annotated genes that are contained in protein-coding exons of the fDA-MP-annotated genes (see Supplementary Figure S1 for details). The coverage distribution is provided for the genes in benchmark set 1.

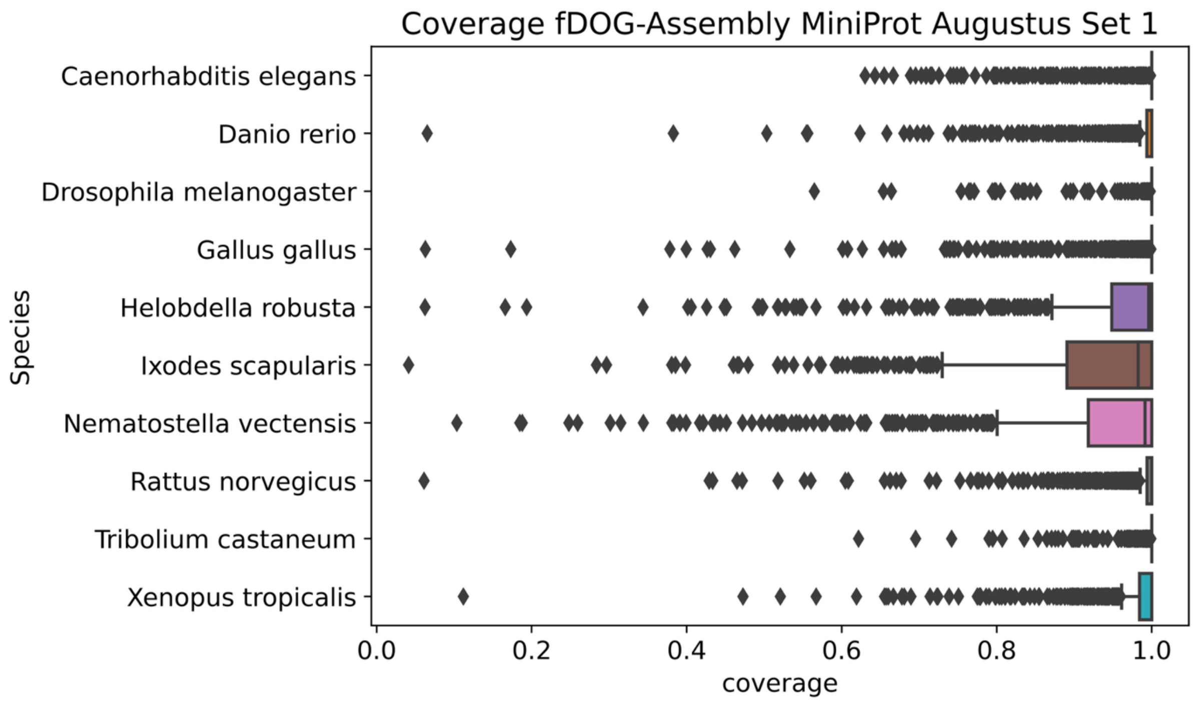

Supplementary Figure S7. Agreement between the fDA-MP$_{Aug}$ gene annotation and the pre-annotated genes in the individual QfO reference species. 'Coverage' denotes the fraction of nucleotides in the protein-coding exons of the pre-annotated genes that are contained in protein-coding exons of the fDA-MP$_{Aug}$-annotated genes (see Supplementary Figure S1 for details). The coverage distribution is provided for the genes in benchmark set 1.

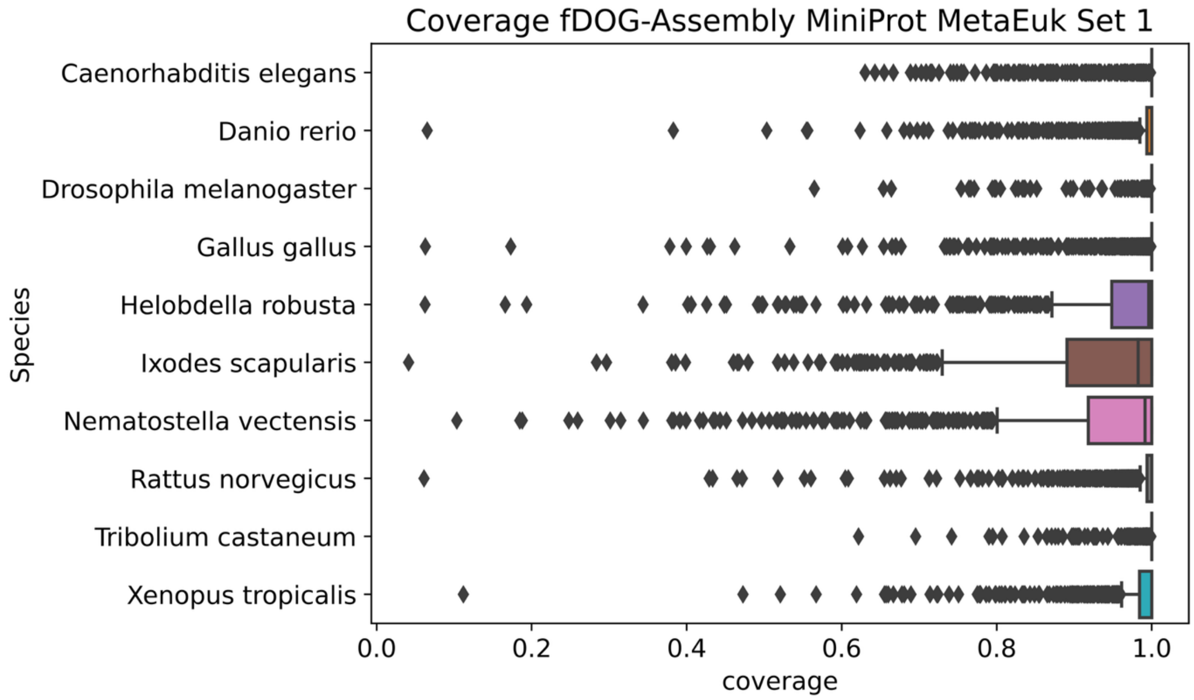

Supplementary Figure S8. Agreement between the fDA-MP$_{ME}$ gene annotation and the pre-annotated genes in the individual QfO reference species. 'Coverage' denotes the fraction of nucleotides in the protein-coding exons of the pre-annotated genes that are contained in protein-coding exons of the fDA-MP$_{ME}$-annotated genes (see Supplementary Figure S1 for details). The coverage distribution is provided for the genes in benchmark set 1.

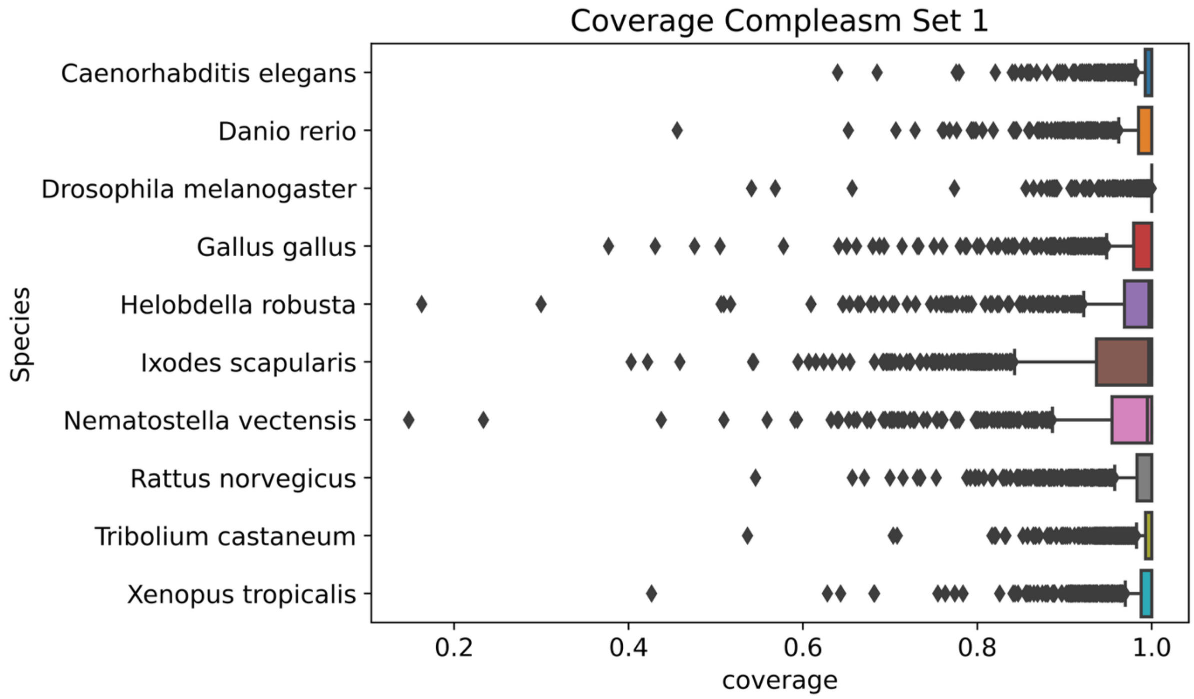

Supplementary Figure S9. Agreement between the Compleasm gene annotation and the pre-annotated genes in the individual QfO reference species. 'Coverage' denotes the fraction of nucleotides in the protein-coding exons of the pre-annotated genes that are contained in protein-coding exons of the Compleasm-annotated genes (see Supplementary Figure S1 for details). The coverage distribution is provided for the genes in benchmark set 1.

Supplementary Figure S10. Overlap of the ortholog assignments made by fDA, Compleasm and BUSCO. The number of orthologs identified in the individual searches is provided in the first column and indicated by the bar. Patterns of stacked filled circles indicate orthologs consistently identified by the respective tools, and the total number per pattern is given in the bar chart. See *Supplementary_Figure_S10_Muelbaier_fDA.pdf*

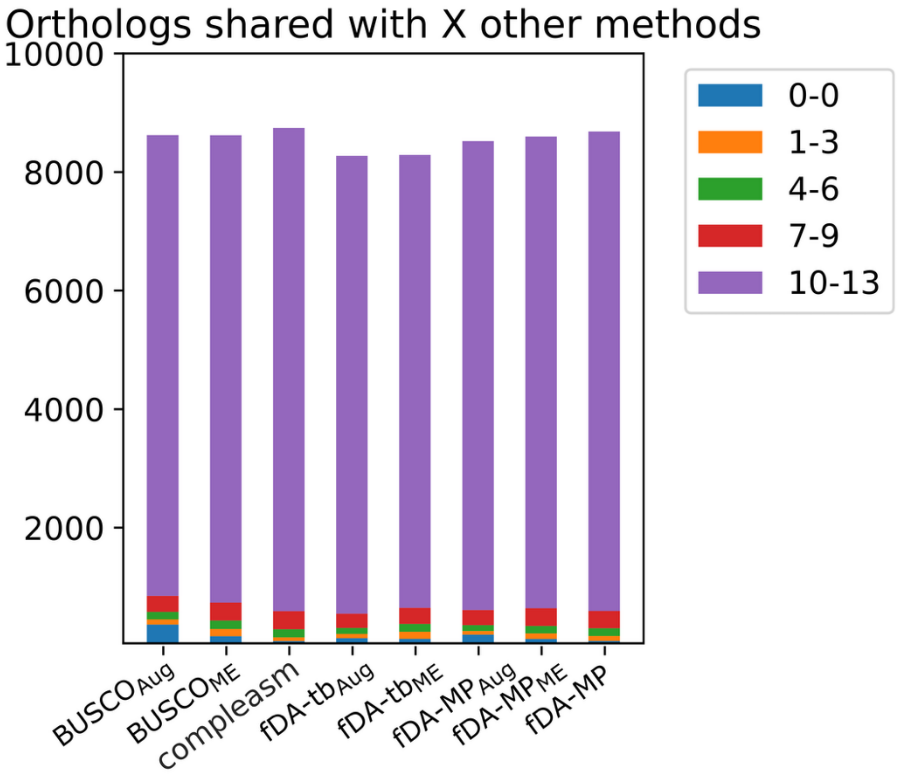

Supplementary Figure S11. Consistency of orthology assignments from assembly-based tools with those from proteome-based tools.
The color code indicates how many of the proteome-based tools support each assignment made by the respective assembly-based tool. Bar heights specify the number of ortholog assignments within each category (linear scale).

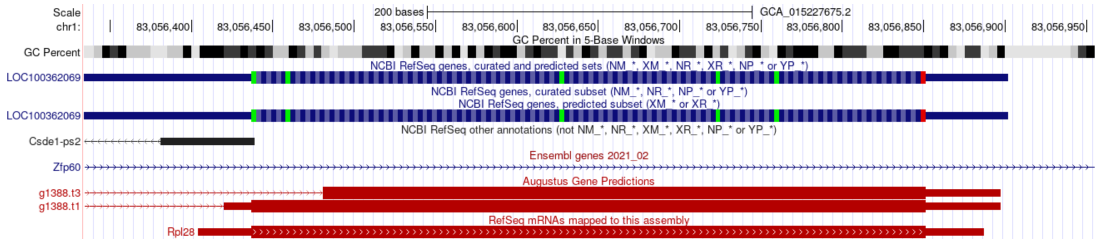

Supplementary Figure S12. The fDA-tb$_{AUG}$ ortholog to the BUSCO gene 613851at33208 overlaps with a curated NCBI RefSeq gene that is not included in the QfO reference proteome. fDA$_{Aug}$ identified an ortholog in *Rattus norvegicus* (GCA_015227675) that closes a gap in the phylogenetic profile that was based on the analysis of the QfO reference proteomes using conventional ortholog search tools. Mapping the identified ortholog to the genome assembly (Augustus gene prediction) reveals that it fully overlaps with an annotated RefSeq protein and a RefSeq mRNA.

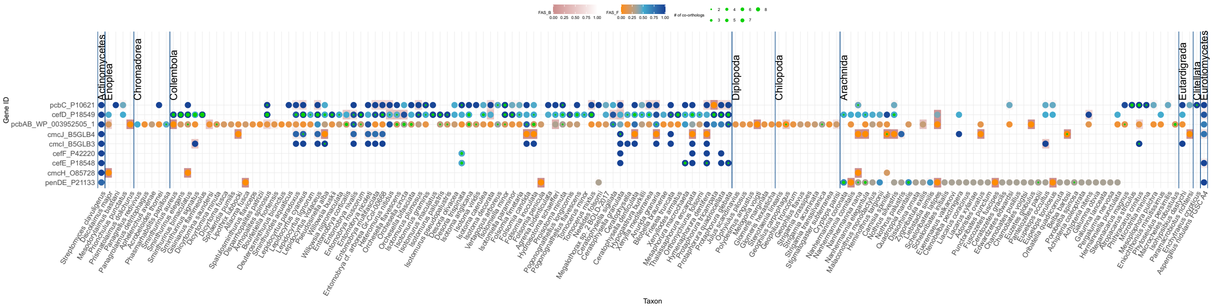

Supplementary Figure S13. Phylogenetic Profiles of genes involved in beta-lactam biosynthesis across soil-living invertebrates. Each row represents the phylogenetic profile for the gene indicated in the first column. The first and the last column represent *Streptomyces clavuligerus*, and *Aspergillus nidulans*, respectively. *S. clavuligerus* served as the reference species for all fDA searches except for penDE, where *A. nidulans* was used. Dots indicate the detection of an ortholog to the respective seed gene in the individual species. The dot color encodes the feature architecture similarity between the seed protein and its ortholog using the seed architecture as reference. The color gradient ranges from blue (high similarity) to orange (low similarity). The cell color encodes the feature architecture similarity between the seed protein and its ortholog using the ortholog architecture as reference. The color gradient ranges from white (high similarity) to pink (low similarity). Green inlays indicate that fDA detected more than one co-ortholog.

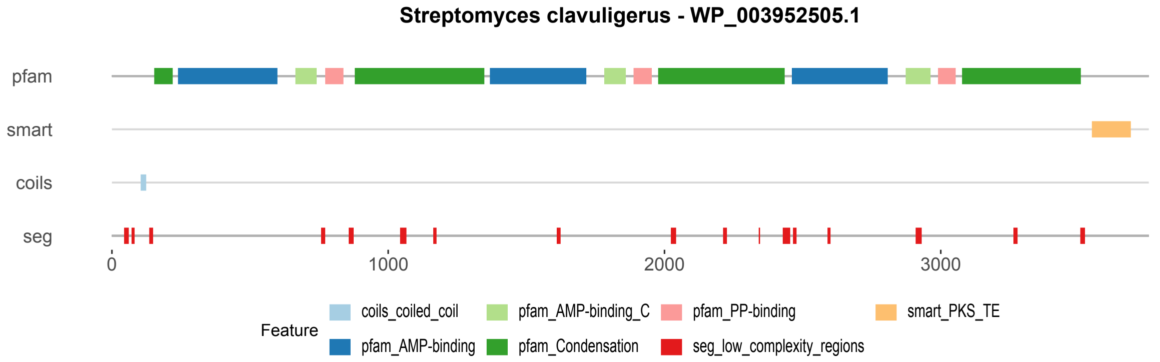

Supplementary Figure S14. Domain Architecture of pcbAB (d-(L-a-Aminoadipyl)-L-Cysteinyl-D-Valine Synthetase) from Streptomyces clavuligerus. Pfam_AMP-binding - PF00501; pfam_AMP-binding_C - PF13193; pfam_Condensation - PF00668; pfam_PP-binding - PF00550; smart_PKS_TE - SM000824
